# Globally learning gene regulatory networks based on hidden atomic regulators from transcriptomic big data

**DOI:** 10.1186/s12864-020-07079-8

**Published:** 2020-10-14

**Authors:** Ming Shi, Sheng Tan, Xin-Ping Xie, Ao Li, Wulin Yang, Tao Zhu, Hong-Qiang Wang

**Affiliations:** 1 MICB Laboratory, Institute of Intelligent Machines, Hefei Institutes of Physical Science, CAS, 350 Shushanghu Road, Hefei, Anhui 230031 P. R. China; 2 Current Address: MOE Key Laboratory of Bioinformatics, Division of Bioinformatics and Center for Synthetic and Systems Biology, TNLIST, Department of Automation, Tsinghua University, Beijing, 100084 China; 3 The CAS Key Laboratory of Innate Immunity and Chronic Disease, Division of Life Sciences and Medicine, University of Science and Technology of China, 96 Jinzhai Road, Hefei, Anhui 230026 P. R. China; 4 School of Mathematics and Physics, Anhui Jianzhu University, 856 Jinzhai Road, Hefei, Anhui 230022 P. R. China; 5 School of Information Science and Technology, University of Science and Technology of China, 96 Jinzhai Road, Hefei, Anhui 230026 P. R. China; 6 Cancer hospital & Anhui Province Key Laboratory of Medical Physics and Technology, Center of Medical Physics and Technology, Hefei Institutes of Physical Science, CAS, 350 Shushanghu Road, Hefei, Anhui 230031 P. R. China

## Abstract

**Background:**

Genes are regulated by various types of regulators and most of them are still unknown or unobserved. Current gene regulatory networks (GRNs) reverse engineering methods often neglect the unknown regulators and infer regulatory relationships in a local and sub-optimal manner.

**Results:**

This paper proposes a global GRNs inference framework based on dictionary learning, named dlGRN. The method intends to learn atomic regulators (ARs) from gene expression data using a modified dictionary learning (DL) algorithm, which reflects the whole gene regulatory system, and predicts the regulation between a known regulator and a target gene in a global regression way. The modified DL algorithm fits the scale-free property of biological network, rendering dlGRN intrinsically discern direct and indirect regulations.

**Conclusions:**

Extensive experimental results on simulation and real-world data demonstrate the effectiveness and efficiency of dlGRN in reverse engineering GRNs. A novel predicted transcription regulation between a TF TFAP2C and an oncogene EGFR was experimentally verified in lung cancer cells. Furthermore, the real application reveals the prevalence of DNA methylation regulation in gene regulatory system. dlGRN can be a standalone tool for GRN inference for its globalization and robustness.

## Background

Gene regulatory networks (GRNs) play fundamental and central roles in response to endogenous or exogenous stimuli for maintaining the viability and plasticity of cells [1, 2]. Although it has been acknowledged that aberrant gene networks can be a key driver of human diseases including cancer, little is known about the GRNs of cancer, which has largely impeded the development of cancer precision medicine [3–5]. In these years, a deluge of omics big data has been generated and accumulated worldwide, which provides an unprecedented opportunity for reverse engineering GRNs in a cost-efficient way [6, 7]. Efficient computational models for inferring GRNs from these omics data are urgently needed theoretically and practically.

Generally, several key issues need to be carefully dealt with in inferring GRNs [7]: 1) Highly complex and heterogeneous networking. Various types of regulations, e.g., transcriptional, methylation or miRNA regulations, are involved and mutually interwoven in GRNs; 2) A large number of regulatory elements or variables unknown or hidden; 3) Discerning indirect and direct interactions; 4) Prior knowledge exploitation or integration of multi-omics data. Broadly speaking, according to the way of modeling transcriptional expression patterns [8], current GRN inference methods can be divided into two categories: parameterized topology paradigm (PTP) and un-parameterized topology paradigm (UPTP). The former attempts to model expression patterns of genes including TFs by parameterizing the topology of the GRNs with various methods [9, 10], such as probabilistic graphical models, ODEs and Petri Nets, while the latter treats each pair or subset of regulators and target genes locally and then assembles them into a complete network [11]. In PTP, the use of generative network models allows to take advantage of prior knowledge and favors a global inference for GRNs recovery. A main disadvantage of the PTP methods, however, is the expensive computational cost raised by the heuristic or greedy search for network parameters in an extremely large space. For example, Gaussian graphical models need to estimate a partial correlation matrix of size at least square of the number of genes [12, 13]. Compared with Gaussian graphical models, Bayesian networks can tell about both the strength and the direction of regulations, leading to a great prevalence in practice [14]. Friedman et al [10] firstly introduced Bayesian networks to reconstruct *S. cerevisiae*’s gene networks. Recently, Siahpirani et al. [15] considered three types of prior biological knowledge, ChIP, motif and knockout, in an integrative Bayesian network for GRNs inference. For more related works, refer to other literature, e.g. [6, 14, 16–18].

In contrast, UPTP methods often make use of similarity measures [19], e.g. Pearson correlation (PC), mutual information (MI)*,* or their variants, to score the confidence of regulations between a pair of genes. For this reason, the resulting GRNs are also called dependency networks. Among the similarity measures, most commonly used is MI for its particular power of modeling complex dependencies [20]. For example, the ARACNE method, proposed by Margolin et al. [21], combined MI with the data procession inequality (DPI) to recover GRNs. Due to the transitive effect of correlations and the limited number of observations, ARACNE tends to be over-sensitive to the high noise in microarray data, often yielding plenty of false positives in practice. To overcome the over-sensitivity, Meyer et al. [22] introduced the maximum relevance/minimum redundancy filter for refinement, and Liu et al. [23] designed another two redundancy reduction algorithms specifically for eliminating weakly indirect and noise-induced regulations respectively. Compared with MI, conditional MI (CMI) can provide a constringent result by calculating the mutual information of two genes conditional on other genes [24]. Recent studies show that direct use of CMI, however, tends to have a too conservative result due to the rigid conditional constraint [25]. To relax the constringency, Zhang et al. [26] developed a new conditional MI, CMI2, for characterizing the causal associations between genes, which alternatively quantifies the conditional mutual information through calculating the Kullback–Leibler divergence. By combining CMI2 with path consistency algorithm, the Zhang’s model can accurately measure the correlations between gene-pairs for keeping synergistic regulations, thus alleviating the underestimation problem of CMI. For these conditional measures, one more big challenge still remains, i.e. the optimal selection problem of conditional genes, due to lack of prior knowledge.

Recently, target gene-centric regression models (TGCR) have attracted increasing attentions for GRNs reconstruction [2, 23, 27]. They mainly rely on regression models, instead of the similarity measures described above, and can favourably bypass the challenging optimal selection problem of conditional genes in conditional correlation models like CMI. Briefly, a TGCR method regresses the expression levels of a target gene on known transcriptional factors (TFs) and reports TFs with non-zero coefficients to be a regulator for the target gene. Many regression models have been explored in this way for GRN inference [6], for example, sparse models including *l*_1_ or *l*_0_ regularized regression [28, 29]. Compared with the pair-wise paradigm above, TGCR can approach a global inference of regulators for a target gene by trying to estimate a global objective. For example, the recently developed GENIE3 [30], which won the DREAM5 network inference challenge, decomposes the reconstruction of a *p*-gene regulatory network into *p* different regression problems. In each of the regression problems, a tree-based ensemble model, Random Forests or Extra-Trees [31], is applied to calculate a local ranking of genes, and the resulting *p* local rankings are finally aggregated to reach a global ranking of all gene pairs.

Dictionary learning (DL) is a recently developed signal restoration model, which finds a dictionary of atomic vectors for a sparse representation of the observed data [32, 33]. Extensive applications in different signal processing fields such as image denoising, audio processing as well as pattern classification, have witnessed the great success of DL in recovering hidden signals [34]. We here develop a DL-based GRN inference framework (dlGRN), which intends to learn a sparse representation of the gene regulatory system via a modified DL algorithm and then makes a global inference of the regulators for a target gene based on the sparse representation, independent of known or observed regulators. We argue that it is the first time to truly globally reverse engineering GRNs with the help of a sparse representation of the regulatory system. We demonstrated the effectiveness and efficiency of the proposed method on synthetic data and real-world data about two model organisms and human lung cancer. A novel predicted regulation of a TF, TRAP2C, on an oncogene, EGFR, was experimentally verified. dlGRN is also versatile to infer DNA methylation regulations besides the most concerned transcriptional regulations.

## Results

### Overview of dlGRN

**Fig. 1:**
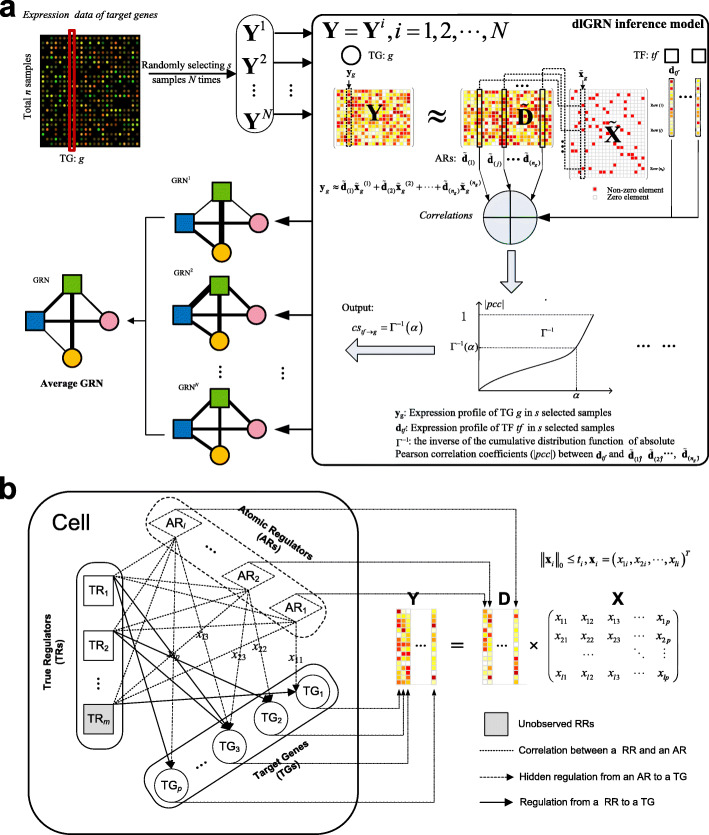
The global inference framework of GRNs (dlGRN). **a** Pipeline of dlGRN. **b** A global gene regulatory model based on ARs (GGRM)

Figure 1a shows the pipeline of dlGRN. The proposed method first decomposes the expression matrix of target genes (TGs) using a modified DL algorithm to uncover atomic regulators (ARs), which as basic regulatory signals reflecting the whole regulatory landscape underpinning the expression data, as shown in Fig. 1b. The modified DL algorithm, called sf*k*-svd, fits the scale-free and sparse property of GRNs. Given a pair of TF *tf*, and TG *g*, dlGRN then estimates Pearson correlations (PCs) between *tf* and the resulting ARs associated with *g* and calculates a confidence score (cs) for the regulation of *tf* on *g* via the inverse function of the cumulative distribution of PCs, as shown in Fig. 1a. The confidence score is meaningful in systems biology and will be robust due to the globalization of ARs. To avoid small sample bias, we also devise a resampling procedure to wrap the inference model and obtain a final GRN as an average network over multiple runs (Fig. 1a).

### Evaluation of the performance of uncovering hidden ARs

**Fig. 2:**
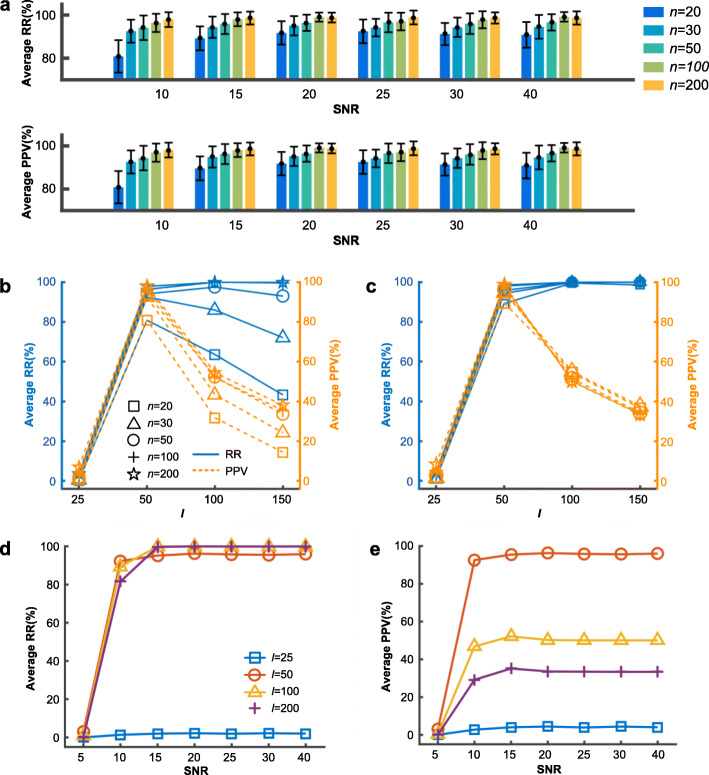
Evaluation of the signal recovery power of dlGRN on Simulation data I. **a** Error barplots of average RRs and average PPVs (*l* = 50). **b**, **c** Changing curves of average RRs and average PPVs with *l* at SNR = 10 (**b**) and 15 (**c**). **d, e** Changing curves of average RRs(**d**) and average PPVs(**e**) over sample sizes with SNR

When applying sf*k*-svd to Simulation data I, we observed that root mean squared errors (RMSEs) gradually decreased and converged within ~ 200 iterations in all the data scenarios (Figs. S1-S5 in S1 Notes), irrespective of the values of *l,* {25, 50, 100 and 150}, suggesting the convergence of the algorithm. With the learned ARs, we calculated RRs and PPVs against the 50 real regulators and averaged them over 20 random data sets in each scenario. Results show that both RRs and PPVs reached a maximum of > 90%, irrespective of sample sizes and noise levels (Fig. 2a), indicating the super power of dlGRN in learning hidden regulatory signals. We observed that the parameter *l* took substantial impact on the power: *l =* 50*,* i.e., the real number of regulators, always led to the highest RRs and PPVs, while *l* > 50 or *l* < 50 decreased PPVs significantly, as shown in Fig. 2 b-c. This may relate to the efficacy of dictionary learning, which can be incomplete or over-complete depending on *l*, and on the other hand, suggests that an optimal *l* tends to be equal to or slightly larger than the real number of regulators. Similar results were obtained in simulation scenarios of *SNR* = 20, 25, 30 and 40 (Fig. S6 in Supplemental material SI Notes). Figure 2(b, c) also reveal that the power increases as the sample size becomes larger, especially when noise is high (Fig. S6). Figure 2d-e visualizes the changes of the average RRs and average PPVs over different samples sizes with *SNR*, showing a trend that the power increases as noise reduces, especially when *l* is large.

### Evaluation of the performance of dlGRN in predicting gene regulations

Results reveals that on Simulation data I, dlGRN achieved higher average AUROCs and AUPRs than four state-of-the-art methods, GENIE3 [30], CLR [35], ARACNe-AP [11] and ARACNE [21] in all the scenarios of sample sizes and noise levels, as shown in Table 1 (and Table S1 in Supplemental material SII Notes). We found that the optimal values of *l* are always around the number of real regulators [36], which is consistent with the pattern of the power of recovering hidden regulatory signals in simulation experiments (Fig. 2). The advantage of dlGRN over previous methods was almost completely kept on the non-linear Simulation data II, shown in Table 2. A main difference is that the maxima of AUROC (83.24%) and AUPR (24.39%) are reached at *l =* 500 (Table S2 in Supplemental material SI Notes), which is far larger than the number (195) of real regulators. This should be related to the increased non-linear complexity in Simulation data II.

**Table 1:** Performances (mean% ± std.% of AUROCs, mean% ± std.% of AUPRs) of different inference methods on Simulation data I (*n* = 20, SNR = 10, 20 and 30). Best results for each SNR case are in bold

METHOD	SNR = 10	SNR = 20	SNR = 30
GENIE3	81.13 ± 0.49, 53.83 ± 0.77	82.05 ± 0.51, 56.11 ± 0.61	81.89 ± 0.51, 56.04 ± 0.78
CLR	81.04 ± 0.29, 57.66 ± 0.42	81.95 ± 0.36, 59.80 ± 0.53	81.74 ± 0.44, 59.50 ± 0.69
ARACNe-AP	62.56 ± 1.45, 13.68 ± 5.37	64.17 ± 0.61, 18.63 ± 3.77	64.13 ± 1.32, 19.17 ± 7.03
ARACNE	81.84 ± 0.43, 55.20 ± 0.53	82.65 ± 0.41, 57.18 ± 0.73	82.55 ± 0.62, 56.37 ± 1.10
dlGRN (*l* = 25)	88.21 ± 0.51, 65.49 ± 1.10	90.05 ± 0.67, 70.10 ± 1.49	90.62 ± 0.41, 71.05 ± 0.89
dlGRN (*l* = 50)	**91.06 ± 0.31, 77.77 ± 1.00**	96.10 ± 0.42, **89.88 ± 1.12**	97.45 ± 0.29, **92.11 ± 1.48**
dlGRN (*l* = 100)	90.48 ± 0.45, 75.16 ± 1.06	**96.23 ± 0.65**, 89.18 ± 1.39	**97.73 ± 0.32**, 92.06 ± 0.22
dlGRN (*l* = 150)	90.40 ± 0.45, 75.86 ± 0.98	95.96 ± 0.32, 87.73 ± 0.41	97.54 ± 0.46, 91.44 ± 1.13

**Table 2:** Results (AUROCs%, AUPRs%) of different methods on simulation data II, two real-world model organism data sets and three lung cancer data sets GSE32863, GSE10072, GSE7670. Best values for each data set are in bold

Data sets	GENIE3	CLR	ARACNe-AP	ARACNE	dlGRN
Simulation data II	81.50, **28.36**	74.34, 22.63	68.19, 15.59	75.72, 19.12	**83.24,** 24.39
*E. coli*	**71.67, 2.11**	58.72, 1.12	56.55, 0.61	61.66, 0.80	68.77, 1.69
*S. cerevisiae*	52.94, 0.31	52.43, 0.22	51.64, 0.02	53.01, 0.22	**54.49, 0.41**
GSE32863	52.67, 5.81	51.77, 5.52	51.09, 5.60	52.36, 5.73	**54.84, 6.40**
GSE10072	51.84, 5.54	51.51, 5.43	51.17, 5.42	51.28, 5.40	**52.52, 5.93**
GSE7670	53.17, 5.90	51.74, 5.58	51.64, 5.62	51.04, 5.58	**53.59, 6.29**

**Fig. 3:**
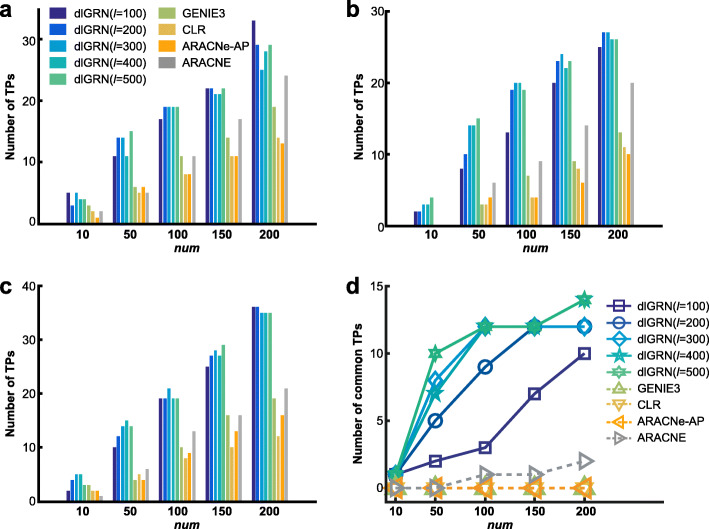
True positives comparison on three lung cancer datasets. Numbers of true positives (TPs) in most highly scored *num* = 10, 50, 100, 150 and 200 regulations by dlGRNs and previous methods (GENIE3, CLR, ARACNe-AP and ARCNE) on three lung cancer data sets GSE32863 (**a**), GSE10072 (**b**), GSE7670 (**c**) and across these data sets (**d**)

For the real two model organisms and three LUAD data sets, AUROCs and AUPRs were calculated against the corresponding experimentally-validated TF-target regulations, respectively. Results reveal that for the *S. cerevisiae* data set and all the three lung cancer data sets, dlGRN still achieved higher AUROCs and AUPRs than those of the four previous methods and competitive results for the *E. coli* data set, as shown in Table 2. For each of the three lung cancer data sets, we further sorted the predicted regulations in a decreasing order of *cs* and counted the numbers of true positives in the top *num* = 10, 50, 100, 150 and 200 for each method, finding that dlGRN called most true positives on all the three data sets and most common true positives, regardless of *num*, as shown in Fig. 3. Taken together, these results suggest the superior power of dlGRN in recovering regulations over state-of-the-art methods.

### The inferred GRNs by dlGRN are globally scale-free, whose nodes tend to locally cluster

**Fig. 4:**
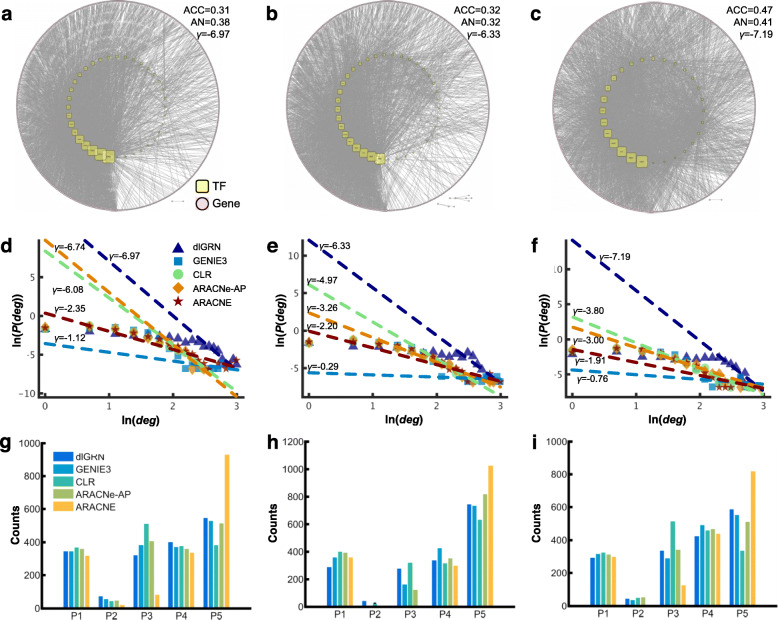
Topological analysis of the reconstructed 2677-link GRNs by dlGRN and the four previous methods. **a**-**c** Topology of GRNs inferred by dlGRN on data sets, GSE32863 (**a**), GSE10072 (**b**) and GSE7670 (**c**). Node sizes are proportional to the connectivity. *γ* : slope of the fitted power-law curve; *AN*: Average number of correctly called TFs *per* target gene; ACC: average clustering coefficient. **d**-**f** Distributions of the log-transformed degrees of nodes in the GRNs on data sets, GSE32863 (**d**), GSE10072 (**e**) and GSE7670 (**f**). **g**-**i** Counts of each CRS pattern (P1-P5) predicted on data sets GSE32863 (**g**), GSE10072 (**h**) and GSE7670 (**i**)

Aberrant gene networks can drive the development of complex diseases such as cancer [37]. Following the known 2677 TF-target regulations, we then selected 2677 TF-target pairs with top *cs* by each method and built GRNs for LUAD on each of the three lung cancer data sets (Fig. 4a-c and Fig. S7 in Supplemental material SI Notes). The *ln*-transformed distributions of the degree of nodes in the resulting GRNs are examined (Fig. 4d-f). Following the power-law property, i.e., ln(P (*deg*)) ~ *γ* ln (*deg*), where *P (deg)* represents the likelihood that a gene has a degree of *deg*, we found that the GRNs obtained by dlGRN have *γ* = − 6.97, − 6.33 and − 7.19 for the three data sets, GSE32863, GSE10072 and GSE7670, respectively, whose absolute values are larger than those by previous methods, indicating more sparse topological structures. In real-life networks, nodes tend to form tightly knit groups with a relatively high density of connectivity [38, 39]. We calculated the average cluster coefficients (ACCs) of these GRNs, finding that the GRNs by dlGRN had larger ACCs (0.31, 0.32, 0.47 for GSE32863, GSE10072 and GSE7670 data sets respectively) than those by all the previous methods on all the three data sets. We further compared the numbers of correctly recognized TFs *per* target gene and the average numbers (*AN*) over all target genes among different methods. Results reveal that the GRNs by dlGRN had *AN* = 0.38, 0.32 and 0.41 for GSE32863, GSE10072 and GSE7670 data sets respectively, as (Fig. 4a-c), which are larger than those by the four previous methods, confirming the higher sensitivity of dlGRN in recognizing regulations. Furthermore, Venn diagrams of the three sets of 2677 links for different methods (Fig. S8 in Supplemental material SI Notes) reveal that dlGRN resulted in significantly more shared links (484) than other methods (*p*-value< 0.001), suggesting the better reproducibility and consistency of GRNs by dlGRN.

**Fig. 5:**
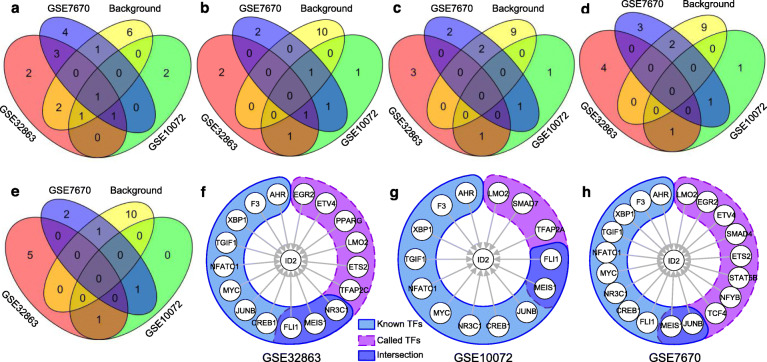
Results of TF regulation inference for target gene ID2 on the three lung cancer datasets (GSE32863, GSE10072 and GSE7670) by different methods. **a**-**e** Venn diagrams of TFs between the background network and the three inferred GRNs by dlGRN (**a**) and four previous methods, GENIE3 (**b**), CLR (**c**), ARACNe-AP (**d**) and ARACNE (**e**). **f**-**h** Known and called TFs of ID2 by dlGRN on data sets, GSE32863 (**f**), GSE10072 (**g**) and GSE7670 (**h**)

Literature survey shows that many predicted TFs by dlGRN for each target gene were previously reported. Take as an example the target gene “ID2”, whose encoding protein belongs to the inhibitor of DNA binding family with a helix-loop-helix (HLH) domain and plays important roles in cell proliferation, differentiation and angiogenesis [40]. Among the 11 experimentally-verified TFs for ID2, 4, 2 and 2 were successfully predicted by dlGRN on the three data sets, GSE32863, GSE10072 and GSE7670, respectively, all more than those by the four previous methods, as shown in Fig. 5a-e. Especially, dlGRN correctly called a known TF of ID2 (MEIS1) simultaneously on the three data sets, but the four previous methods none. Among other regulators predicted by dlGRN, CREB1, missed by all the four previous methods (Fig. 5f-h and Fig. S9 in Supplemental material SI Notes), has been previously reported to regulate ID2 in [41]. For EGR2, Kim et al. [42] experimentally observed that EGR2 transactivates ID2 by binding to the promoter of ID2 and knockdown of EGR2 represses ID2 gene expression in osteoclast-lineage cells. Both ETS2 and ETV4 encode proteins with ETS-domain which can bind to the gene family of IDs [43]. Both SMAD4 and SMAD7 are members of SMAD family, which have previously reported to suppress the expression of ID2 in tumorigenesis [44, 45]. STAT5B is one of two STAT5 TFs from the STAT family, playing important roles in apoptosis and TCR signalling. Li et al. [46] observed that STAT5 proteins regulated ID2 transcription by recruiting STAT5B in a *cis*-regulatory element to the ID2 promoter in dendritic cells. Furthermore, Sun et al. [47] reported that STAT5 stimulates the expression of ID2 to control the CD103^+^ DC production and the pDC inhibition.

### dlGRN is intrinsically distinctive of direct and indirect regulations

Cascade regulatory structure (CRS) is a basic type of regulatory motifs in GRNs [48], where gene A, for example, regulates gene B and gene B subsequently regulates gene C, denoted by A → B → C. In other words, gene A indirectly regulates gene C via two direct regulations. Due to the transitive effect of correlations, current methods often fail to infer CRS completely correctly. The background 2677-link networks of the lung cancer data contain totally 6678 CRSs, against which we investigated how dlGRN distinguishes direct and indirect regulations. Hypothetically, a CRS may be recovered in five patterns (Fig. S10 in Supplemental material SI Notes): Pattern 1 (P1), which wrongly calls a direct regulation between A and C, Pattern 2 (P2), which recovers the CRS completely correctly, and the rest three patterns, named P3, P4 and P5, which correctly recognize the indirect regulation between A and C but miss direct regulations, A → B, B → C, or both, respectively. Figure 4g-i compares the numbers of the five patterns detected by dlGRN and the four previous methods on the three data sets, showing that dlGRN completely correctly called most CRSs (P2) with least recognition errors of indirect regulations (P1) on almost all the data sets. ARACNe missed most direct regulations (P5) on almost all the three data sets, which may be related to the over-trimming of links by DPI. These results suggest that dlGRN is intrinsically distinctive of direct and indirect regulations due to the modeling globalization.

### A novel predicted transcriptional regulation of TFAP2C on EGFR in lung cancer

**Fig. 6:**
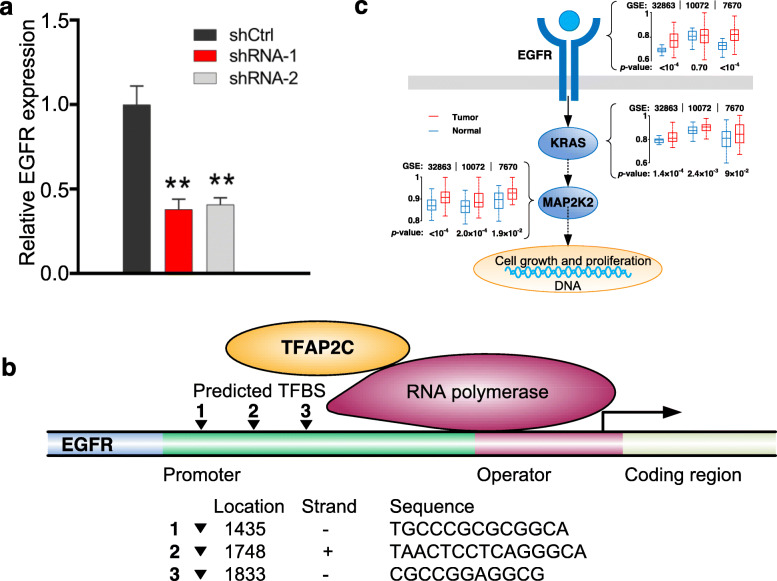
Experimental verification of TFAP2C regulating EGFR in A549 cancer cells. **a** Relative expression levels of EGFR with or without knockdown of TFAP2C. “**” mean *p*-values< 0.01. **b** Potential transcriptional regulation mechanism of TFAP2C on EGFR. **c** Comparison of expression of genes along onco-signalling pathway activated by EGFR between tumor and normal tissues in the three data sets

Considering that EGFR is one of hottest onco-genes in lung cancer, we looked into the predicted TFs for EGFR by dlGRN on the three lung cancer data sets. We averaged the resulting *cs* over the three data sets for each of the 55 known TFs (Supplemental material SIII Notes), and found that two TFs, LMO2 and TFAP2C, not recorded as TFs of EGFR in the UCSC and TRED databases (April, 2017), are with highest average *cs* (0.36 and 0.33), suggesting a high likelihood of regulating EGFR. To experimentally verify the predictions, we searched for the transcription factor binding sites (TFBSs) of the two TFs to the promoter of EGFR using the online JASPAR tool (http://jaspar.genereg.net/), finding 93 TFBSs for TFAP2C but none for LMO2. Based on the 93 TFBSs, we conducted TFAP2C siRNA knockdown experiments on lung cancer cell A549. As a result, we observed that EGFR significantly (*p*-value< 0.01) depressed its expression after knockdown of TFAP2C in the two repeats (Fig. 6a). Similar depression has been previously observed in luminal breast cancers [49]. A potential regulatory mechanism of EGFR by TFAP2C may be via three most highly scored TFBSs predicted by JASPAR, as shown in Fig. 6b. According to KEGG pathway database (https://www.genome.jp/kegg/pathway.html), activated EGFR can lead to cell growth and proliferation via RAS-MAPK signalling pathway. We found that many genes along the RAS-MAPK signalling pathway, e.g., RAS and MAP 2 K1, significantly over-expressed in tumors compared with normal tissues in the three lung cancer data sets (Fig. 6c).

### dlGRN reveals the prevalence of DNA methylation regulation in gene regulatory system

**Fig. 7:**
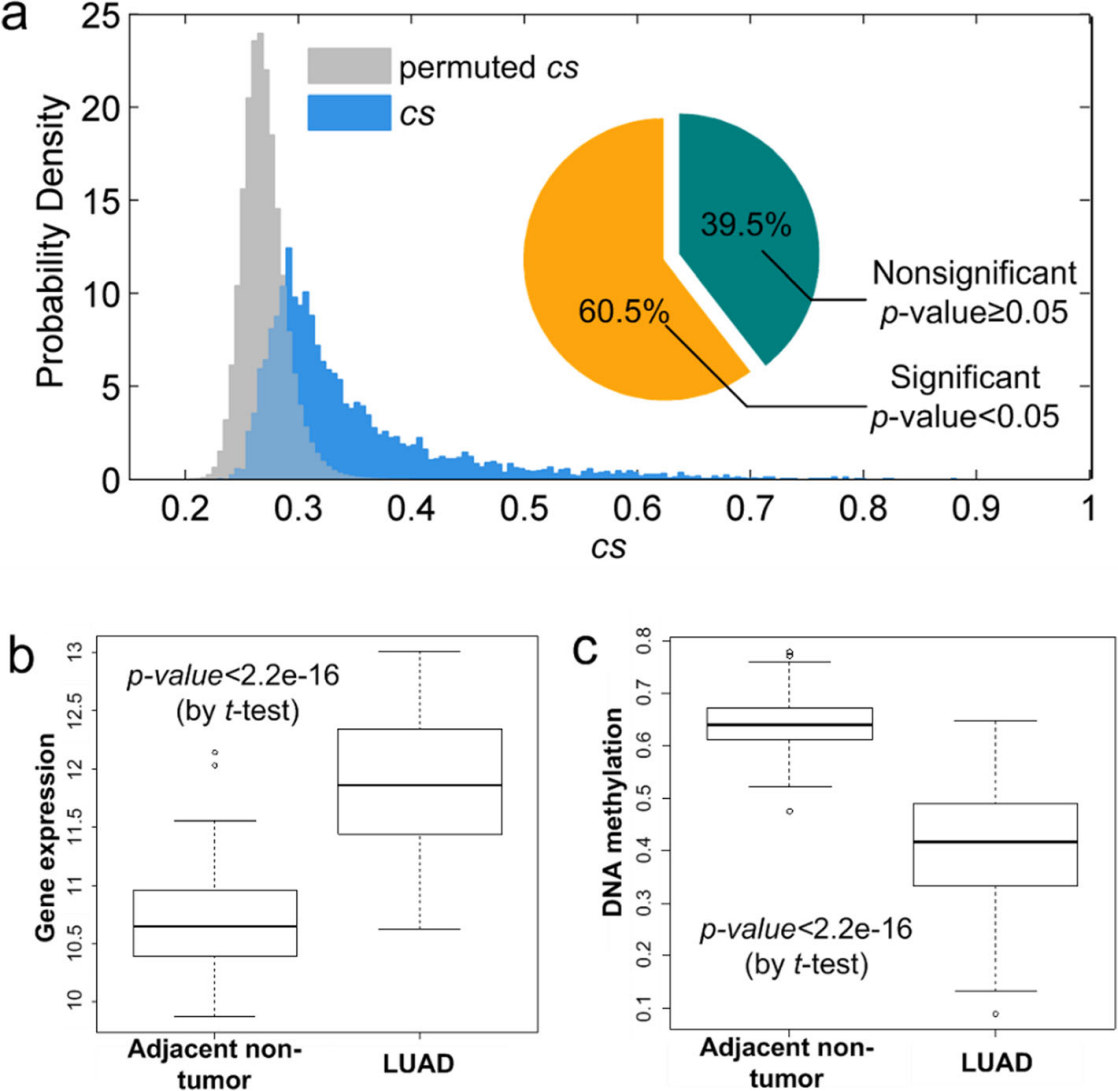
DNA methylation regulation frequently occurs in cellular activity. **a** Comparison of probabilistic density between permutated and observed methylation regulation scores. **b**, **c** Boxplots of expression and DNA methylation of gene RAB25 in LUAD and adjacent normal tissues on data sets GSE32861 and GSE32863

By replacing the expression levels of TFs with DNA methylation levels, dlGRN can be used to infer DNA methylation regulation of genes. For the lung cancer data set, GSE32863, Selamat et al. [50] monitored the DNA methylation profiles of the 116 samples at the same time. We downloaded the DNA methylation data (GSE32861) from GEO and applied dlGRN to jointly analyze it with the expression data, GSE32863. Results reveal that a considerable proportion of genes (60.5%) are significantly methylation-regulated at an ad hoc *p*-value cutoff of 0.05 (by a permutation test described in Supplemental material SI Notes), as shown in Fig. 7a (and Supplemental material SIV Notes). This coincides with the indispensible roles of DNA methylation in cellular activity [36]. Many of the inferred methylation regulations have been previously observed as hypo- or hyper methylations in cancer (Table S3 in Supplemental material SI Notes). Take gene “RAB25” (*cs* = 0.6976, *p*-value<1*e*-3) as example. The gene belongs to the RAS superfamily of small guanosine triphosphatase (GTPase), which regulates tumor progression and aggressiveness during tumorigenesis. Figure 7b-c boxplots the expression and methylation levels of RAB25 in tumor and adjacent non-tumor tissues, showing that RAB25 is both significantly up-expressed (*p*-value< 2.2e-16) and significantly down-methylated (*p*-value< 2.2e-16) in the LUAD. Correlation analysis confirms that RAB25 expression is significantly negatively correlated with its methylation (Pearson correlation is − 0.67 and *p*-value = 3.76e-16). We reason that the abnormal over-expression of RAB25 in LUAD may be driven by its aberrant hypo-methylation, albeit needs to be experimentally verified.

## Discussion

In this paper, we have proposed a global inference framework for reverse engineering GRNs based on deep learning, i.e. dlGRN. The framework interrogates the gene regulatory system using DL and predicts regulations between TFs and TGs in a global way. Specifically, a modified DL algorithm sf*k*-svd was developed for reliably uncovering ARs which reflect the whole regulatory mechanism. The modified DL algorithm fits the scale-free and sparse property of GRNs. Then, the regulation confidence of a TF on a target gene can be estimated by a correlation analysis between the TF and the ARs associated with the target genes. The use of ARs guarantees the globalization of the regulation inference. A resampling procedure was also designed to avoid sample biases for inference robustness. Experiments on simulation and real data sets show that dlGRN outperforms state-of-the-art methods with higher AUROCs and higher AUPRs in GRN reconstruction.

Discerning indirect and direct regulations is of importance in GRN reconstruction. Previous methods such as similarity criteria often call plenty of spurious direct regulations due to the transitive effect of correlations. In contrast, dlGRN works in a AR-based global way and thus is intrinsically distinctive of direct and indirect regulations, as illustrated in the experiment on lung cancer data (Figs. S8, S9), where dlGRN correctly recognized most CRS modules with least errors on almost all the three data sets.

We experimentally verified a novel predicted regulation, i.e., the regulation of TF TFAP2C on a hot once-gene EGFR, in lung cancer cell A549 and conceived a potential three TFBSs molecular mechanism. Over-expressed EGFR can stimulate cell growth and proliferation via RAS-MAPK signalling pathway. Many genes along the pathway, e.g.*,* RAS and MAP 2 K1, were observed to be over-expressed in tumors in the three lung cancer data sets (Fig. 6c), confirming the downstream of tumor signals trigged by the abnormal TFAP2C-EGFR regulation. In addition, we also revealed the prevalence of DNA methylation regulation in gene regulatory system. Considering the pressing need of understanding GRNs in cells, we envision that our approach will be very useful and promise broad applications in biological and medical research.

Despite the success of recovering TF/DNA methylation regulations, gene regulatory system is complex and involves various types of expression regulations, for example, histone modification and miRNA degradation, which regulate target genes in different ways and may need more specific reverse engineering models. We also notice that TFs preferentially bind to a certain target sequence, and searching for that sequence or similar patterns in the regulatory regions of the target genes may help improve dlGRN. Future work will be addressing these issues for better performance.

## Methods

### A global model of gene regulatory systems (GGRM)

In cells, gene expression can be regulated and mediated in concert by various types of regulatory factors, such as TFs, microRNAs or epigenetic states. We hypothesize that the expression levels of a target gene are collectively shaped by a handful of basic regulators in a weighted linear way. Considering that plenty of regulators are unknown or unobserved, we intend to interrogate the whole regulatory mechanism by mining as many hidden basic regulatory signals as possible via dictionary learning, as shown in Fig. 1b. Theoretically, the resulting regulatory signals, referred to as atomic regulators (ARs), can represent all possible regulatory factors, such as TFs, microRNAs, epigenetic statuses, or even combinational regulatory modules. Mathematically, let $\mathbf{Y}\in {\mathtt{R}}^{n\times p}$ denote an observed expression matrix of *p* target genes in *n* samples, we reformulate Y as

1 $$ \mathbf{Y}=\mathbf{DX}+\varepsilon $$

where $\mathbf{D}\in {\mathtt{R}}^{n\times l}$ represents the regulatory dictionary matrix of *l* ARs across *n* samples, incomplete or over-complete; $\mathbf{X}\in {\mathtt{R}}^{l\times p}$ represents the sparse regulation coefficient matrix of the *l* ARs on target genes, of which element *x*_*ij*_ represents the regulation effect of the *i*-th AR to the *j*-th target gene; **ε** is a random white noise subjecting to an *i.i.d* Gaussian distribution with mean of zero. The learned AR dictionary reflects a surrogate of the regulatory mechanisms underlying **Y**. The number of ARs (*l*) is an important parameter to learn all the ARs behind the expression data. However, no exact guidance exists for choosing the parameter in practice. Theoretically, the parameter should be large enough for a comprehensive regulatory picture, for example, at least larger than the number of known or real regulators, while not too large values are necessary to avoid overfitting. Empirically, one can try different values and choose the best one.

Considering the sparse and power-law property of GRNs, we solve the model [1] by simultaneously optimizing **D** and **X** under a scale-free sparsity constraint:

2 $$ \left(\hat{\mathbf{D}},\hat{\mathbf{X}}\right)=\arg \underset{\mathbf{D},\mathbf{X}}{\min }{\left\Vert \mathbf{Y}-\mathbf{DX}\right\Vert}_2^2\kern1em \mathrm{s}.\mathrm{t}.\kern1em {\left\Vert {\mathbf{x}}_i\right\Vert}_0\le {t}_i,i=1,\dots, p $$

where **x**_*i*_ is the *i*-th column of **X** and *t*_*i*_ is a prior positive constant, referred to as scale-free sparsity parameter, that specifies the upper boundary of the number of ARs for the *i*-th target gene. We set *t*_*i*_ by randomly sampling from a distribution *P*(*t*_*i*_) ∝ *t*_*i*_^−*λ*^ (λ = 2–4 and *t*_*i*_= 2–6). The optimization [2] guarantees the sparsity of the resulting GRNs and makes it under control in network structure. However, the objective function is not convex on both **D** and **X** together and is algorithmically NP-hard. Mathematically, for such optimization problems, no solutions are immediately available and local minima is often desirable in practice [51]. We then developed a modified *k*-SVD algorithm, named scale-free-constrained *k*-SVD (sf*k*-svd), to approximate a local solution for the optimization problem [2]. Briefly speaking, the algorithm repeats two steps, i.e., sparse approximation and dictionary update, until the error converges (See Section 1 in S1 Notes for details).

There are many methods that can be used to analyze the latent regulatory signals, such as PCA [52], ICA [53] or NCA [54]. However, these methods either impose strict statistical properties for learned latent regulators, e.g. orthogonality (PCA) and independence (ICA), or strongly needs priori connectivity information, e.g. NCA, which makes them not suitable and scalable for large scale biological systems inference, especially with limited number of samples [55, 56]. Compared with these methods mentioned above, *k*-SVD-like dictionary learning methods are promising, because they hardly impose none statistical properties on the atomic regulators to be mined except the sparseness of the inferred network structure which is in coordination with the real-world GRN structure.

### Inferring regulatory relationships based on ARs

Let $\left(\hat{\mathbf{D}},\hat{\mathbf{X}}\right)$ represent a solution for the GGRM. For the *g*-th target gene, assume that the *g*-th column ${\hat{\mathbf{x}}}_g$ of $\hat{\mathbf{X}}$ has *n*_*g*_ non-zero elements with subscripts (1), (2), …, (*n*_*g*_), we can have *n*_*g*_ ARs that are associated with the target gene,

3 $$ {S}_{AR}=\left\{{\hat{\mathbf{d}}}_{(1)},{\hat{\mathbf{d}}}_{(2)},\dots, {\hat{\mathbf{d}}}_{\left({n}_g\right)}\right\} $$

where ${\hat{\mathbf{d}}}_{(i)}$ is the (*i*)-th column of $\hat{\mathbf{D}}$. For a given TF *tf* with an expression profile **d**_*tf*_, we then assess how it regulates *g* as follows: First, calculate Pearson correlation coefficients (pcc) between *tf* and each AR. Note that one can use Spearman correlation for non-linear association. Second, estimate the regulation confidence score (*cs*) as

4 $$ {cs}_{tf\to g}={\Gamma}^{-1}\left(\alpha \right) $$

where Γ^−1^ represents the inverse function of the cumulative distribution of |pcc| and 0 ≤ *α* ≤ 1 is a quantile cutoff (*α* = 0.9 as default). Larger *α*s lead to more sensitive results. Figure 1a illustrates the inference procedure.

### A resampling procedure

Considering that resampling can relieve sample bias in machine learning, especially when sample size is small or moderate [57], we also devise a resampling procedure for more reliable inference: 1) Randomly selecting a subset of *s* samples from the total samples without replacement and running dlGRN with the *s* samples; 2) Repeating 1) *N* times to obtain *N cs* by [4] for each pair of regulators and target genes; 3) Averaging the resulting *N cs* as final results. Specifically, we set *s* = 25% × *n* and *N* = 2 × *n* as default. The pseudo code of the proposed GRN inference approach dlGRN can be listed below: 
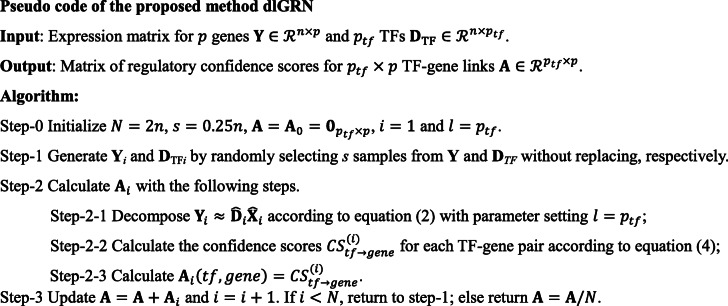


### Parameters of dlGRN

In the proposed GGRM, the parameter *l* represents the number of atomic regulators (ARs) and should approximate to the number of real-world regulators, including TFs, microRNAs and DNA methylation. Theoretically speaking, the value of *l* needs to be estimated based on the biological priors of the organism from which the transcriptomic data was collected. In our context, the value of *l* was set to range around the number of known regulators of genes in the dataset for fully demonstrating the performance of dlGRN. The parameter *t*_*i*_ is a small positive constant to constrain the maximum *l*_0_-norm of the *i*-th regulatory coefficient vector. The resampling procedure makes the inference results insensitive to the selection of *t*_*i*_ within a limited scope [29]. In our context, *t*_*i*_ was set to range in 2–6 in all data scenarios.

### Measures for method evaluation

We adopted two measures, i.e. recovery rate (RR) and positive predictive value (PPV), to evaluate the performance of recovering ARs from gene expression data. Another two measures, i.e. area under receiver operating characteristic curve (AUROC) and area under precision–recall curve (AUPR), were used to assess the performance of detecting regulatory relationships [58]. See Supplemental material S1 Notes for details of these measures.

### Datasets

#### Two simulation data sets

Simulation data I mimic a linear regulatory system with background networks following the sparse and scale-free property, consisting of *p =* 1500 target genes and *k* = 50 regulators. Totally, 30 data scenarios were considered: six noise levels times five sample sizes (See Supplemental material SI Notes for details of Simulation data I generation). Simulation data II were downloaded from the DREAM5 project (http://www.the-dream-project.org/), which are used to mimic a non-linear regulatory system with a background network drawn from known transcriptional regulatory networks of *Yeast Strain*s. The data sets consist of the expression profiles of 1548 target genes and 195 TFs in 805 samples. See the literature [59] for more details of Simulation data II.

#### Five real data sets

First two data sets come from two model organisms, *E. coli* and *S. cerevisiae,* which consist of the expression profiles of 4511 target genes and 334 TFs in 805 samples and the expression profiles of 5950 target genes and 333 TFs in 536 samples, respectively. For the two data sets, 2066 and 3940 experimentally verified TF–TG regulations were collected from the literature [60–62] as silver standard, respectively. Three human lung adenocarcinoma (LUAD) transcriptional data sets, GSE32863, GSE10072 and GSE7670, were downloaded from GEO database and preprocessed (Supplemental material SI Notes) to have the expression levels of 4771 genes in 116, 107, and 54 samples, respectively. For the lung cancer data sets, 2677 TF-target genes regulations were collected from the UCSC database [63] and TRED database [64] (on April 1, 2017) as silver standard.

### Cell culture

Human lung cancer cell A549 was purchased from American Type Culture Collection and cultured in DMEM medium supplemented with 10% fetal bovine serum (Biological Industries, Israel). Cells were cultured in a 37 °C humidified atmosphere of 5% CO2 and planted in a 6-well plate after the cell states became well.

### RNA extraction and quantitative real time PCR

Cancer cells were transfected with siRNAs and negative controls with liposome (lip3000 in our experiments) for 48 h. After the medium was removed, cancer cells were washed 3 times with PBS. Total mRNAs from cultured cells were extracted using Trizol (Invitrogen, UCA) according to the manufacture’s instructions. cDNA was synthesized using the HiScript II 1st Strand cDNA Synthesis Kit (Vazyme Biotech, China), and the expression levels of mRNAs were quantified using ChamQ SYBR Color qPCR Master Mix (Vazyme Biotech, China). Quantitative Real Time PCR was performed using the Bio-Rad CFX Real-time PCR system (Bio-Rad, USA). Statistical comparison of the two groups each with triplicates was conducted using Student’s t-test. Statistically significances were calculated and indicated. *: *P* < 0.05, **: *P* < 0.01.

## Supplementary information

**Additional file 1: SI Notes**. **SII Notes**. **SIII Notes**. **SIV Notes**.

## Data Availability

The source code of dlGRN is available freely at https://github.com/shiming23/dlGRN. The lung cancer data sets are publicly available from Gene Expression Omnibus (GEO) with accession number GSE32863, GSE10072, GSE7670 and GSE32861. The two real model organism data sets (E. coli and S. cerevisiae) are available from http://www.the-dream-project.org/. All other data that support the results of this study are available from the corresponding author upon request.

## References

[1] Gerstein MB, Kundaje A, Hariharan M, Landt SG, Yan KK, Cheng C (2012). Architecture of the human regulatory network derived from ENCODE data. Nature..

[2] Yang AP, Liu LG, Chen MM, Liu F, You H, Liu L (2019). Integrated analysis of 10 lymphoma datasets identifies E2F8 as a key regulator in Burkitt's lymphoma and mantle cell lymphoma. Am J Transl Res.

[3] Barabási A-L, Gulbahce N, Loscalzo J (2010). Network medicine: a network-based approach to human disease. Nat Rev Genet.

[4] Duan Y, Tan Z, Yang M, Li J, Liu C, Wang C (2019). PC-3-Derived Exosomes Inhibit Osteoclast Differentiation by Downregulating miR-214 and Blocking NF-κB Signaling Pathway. Biomed Res Int.

[5] Zhang D, Xia J (2020). Somatic synonymous mutations in regulatory elements contribute to the genetic aetiology of melanoma. BMC Med Genet.

[6] Marbach D, Costello JC, Kuffner R, Vega NM, Prill RJ, Camacho DM (2012). Wisdom of crowds for robust gene network inference. Nat Methods.

[7] Belliveau NM, Barnes SL, Ireland WT, Jones DL, Sweredoski MJ, Moradian A (2018). Systematic approach for dissecting the molecular mechanisms of transcriptional regulation in bacteria. Proc Natl Acad Sci.

[8] Kuffner R, Petri T, Tavakkolkhah P, Windhager L, Zimmer R (2012). Inferring gene regulatory networks by ANOVA. Bioinformatics..

[9] Whittaker J. Graphical Models in Applied Multivariate Statistics1990 4/1/1990.

[10] Friedman N, Linial M, Nachman I, Pe'er D (2000). Using Bayesian networks to analyze expression data. J Comput Biol.

[11] Lachmann A, Giorgi FM, Lopez G, Califano A (2016). ARACNe-AP: gene network reverse engineering through adaptive partitioning inference of mutual information. Bioinformatics..

[12] Ma S, Gong Q, Bohnert HJ (2007). An Arabidopsis gene network based on the graphical Gaussian model. Genome Res.

[13] Tian D, Gu Q, Ma J (2016). Identifying gene regulatory network rewiring using latent differential graphical models. Nucleic Acids Res.

[14] Gendelman R, Xing H, Mirzoeva OK, Sarde P, Curtis C, Feiler HS (2017). Bayesian network inference modeling identifies TRIB1 as a novel regulator of cell-cycle progression and survival in Cancer cells. Cancer Res.

[15] Siahpirani AF, Roy S (2017). A prior-based integrative framework for functional transcriptional regulatory network inference. Nucleic Acids Res.

[16] Luo Y, Mao C, Yang Y, Wang F, Ahmad FS, Arnett D (2018). Integrating hypertension phenotype and genotype with hybrid non-negative matrix factorization. Bioinformatics..

[17] Azad AKM, Lawen A, Keith JM (2017). Bayesian model of signal rewiring reveals mechanisms of gene dysregulation in acquired drug resistance in breast cancer. PLoS One.

[18] Liu F, Zhang S-W, Guo W-F, Wei Z-G, Chen L (2016). Inference of gene regulatory network based on local Bayesian networks. PLoS Comput Biol.

[19] Reshef DN, Reshef YA, Finucane HK, Grossman SR, McVean G, Turnbaugh PJ (2011). Detecting novel associations in large data sets. Science..

[20] Cover TM, Thomas JA. Elements of information theory. 2nd ed. New Jersey: Wiley-Interscience; 2006.

[21] Margolin AA, Nemenman I, Basso K, Wiggins C, Stolovitzky G, Dalla Favera R, et al. ARACNE: an algorithm for the reconstruction of gene regulatory networks in a mammalian cellular context. BMC Bioinformatics. 2006;20;7 Suppl 1(Suppl 1):S7. 10.1186/1471-2105-7-S1-S7 PMC1810318 16723010

[22] Meyer PE, Kontos K, Lafitte F, Bontempi G. Information-theoretic inference of large transcriptional regulatory networks. EURASIP J Bioinform Syst Biol. 2007;Article ID:79879. 10.1155/2007/79879 PMC3171353 18354736

[23] Liu W, Zhu W, Liao B, Chen HW, Ren SQ, Cai LJ (2017). Improving gene regulatory network structure using redundancy reduction in the MRNET algorithm. RSC Adv.

[24] Zhao J, Zhou Y, Zhang X, Chen L (2016). Part mutual information for quantifying direct associations in networks. Proc National Acad Sci USA.

[25] Janzing D, Balduzzi D, Grosse-Wentrup M, Schölkopf B (2013). Quantifying causal influences. Ann Stat.

[26] Zhang X, Zhao J, Hao JK, Zhao XM, Chen L (2015). Conditional mutual inclusive information enables accurate quantification of associations in gene regulatory networks. Nucleic Acids Res.

[27] Gao Y, Yurkovich JT, Seo SW, Kabimoldayev I, Dräger A, Chen K, et al. Systematic discovery of uncharacterized transcription factors in *Escherichia coli* K-12 MG1655. Nucleic Acids Research. 2018:gky752-gky. 10.1093/nar/gky752 PMC6237786 30137486

[28] Geeven G, van Kesteren RE, Smit AB, de Gunst MC (2012). Identification of context-specific gene regulatory networks with GEMULA-gene expression modeling using LAsso. Bioinformatics..

[29] Haury AC, Mordelet F, Vera-Licona P, Vert JP (2012). TIGRESS: trustful inference of gene REgulation using stability selection. BMC Syst Biol.

[30] Huynh-Thu VA, Irrthum A, Wehenkel L, Geurts P (2010). Inferring regulatory networks from expression data using tree-based methods. PLoS One.

[31] Yue Z, Chu X, Xia J. PredCID: prediction of driver frameshift indels in human cancer. Brief Bioinform. 2020. 10.1093/bib/bbaa119. 10.1093/bib/bbaa119 32591774

[32] Wang D, Kong S (2014). A classification-oriented dictionary learning model: explicitly learning the particularity and commonality across categories. Pattern Recogn.

[33] Tosic I, Frossard P (2011). Dictionary learning. IEEE Signal Process Mag.

[34] Jiang Z, Lin Z, Davis LS (2013). Label Consistent K-SVD: Learning a discriminative dictionary for recognition. IEEE Trans Pattern Anal Mach Intell.

[35] Faith JJ, Hayete B, Thaden JT, Mogno I, Wierzbowski J, Cottarel G (2007). Large-scale mapping and validation of Escherichia coli transcriptional regulation from a compendium of expression profiles. PLoS Biol.

[36] Das PM, Singal R (2004). DNA methylation and Cancer. J Clin Oncol.

[37] Iorio MV, Ferracin M, Liu CG, Veronese A, Spizzo R, Sabbioni S (2005). MicroRNA gene expression deregulation in human breast cancer. Cancer Res.

[38] Zhou T, Yan G, Wang B-H (2005). Maximal planar networks with large clustering coefficient and power-law degree distribution. Phys Rev E.

[39] Saramäki J, Kivelä M, Onnela J-P, Kaski K, Kertész J (2007). Generalizations of the clustering coefficient to weighted complex networks. Phys Rev E.

[40] Yates PR, Atherton GT, Deed RW, Norton JD, Sharrocks AD (1999). Id helix–loop–helix proteins inhibit nucleoprotein complex formation by the TCF ETS-domain transcription factors. EMBO J.

[41] Qi L, Saberi M, Zmuda E, Wang Y, Altarejos J, Zhang X, et al. Adipocyte CREB Promotes Insulin Resistance in Obesity. Cell Metabolism 9(3):277–86. 10.1016/j.cmet.2009.01.006 PMC2730923 19254572

[42] Kim H-J, Hong JM, Yoon K-A, Kim N, Cho D-W, Choi J-Y (2012). Early growth response 2 negatively modulates osteoclast differentiation through upregulation of id helix–loop–helix proteins. Bone..

[43] Nishimori H, Sasaki Y, Yoshida K, Irifune H, Zembutsu H, Tanaka T (2002). The Id2 gene is a novel target of transcriptional activation by EWS-ETS fusion proteins in Ewing family tumors. Oncogene..

[44] DiVito KA, Simbulan-Rosenthal CM, Chen Y-S, Trabosh VA, Rosenthal DS (2014). Id2, Id3 and Id4 overcome a Smad7-mediated block in tumorigenesis, generating TGF-β-independent melanoma. Carcinogenesis..

[45] Shi Q, Zhong YS, Ren Z, Li QL, Zhou PH, Xu MD (2011). Analysis of the role of the BMP7-Smad4-Id2 signaling pathway in SW480 colorectal carcinoma cells. Mol Med Rep.

[46] Li HS, Yang CY, Nallaparaju KC, Zhang H, Liu Y-J, Goldrath AW (2012). The signal transducers STAT5 and STAT3 control expression of Id2 and E2-2 during dendritic cell development. Blood..

[47] Sun M, Kee BL (2017). Lnc'ing Id2 to ILC1. Immunity..

[48] Marbach D, Prill R, Schaffter T, Mattiussi C, Floreano D, Stolovitzky G (2010). Revealing strengths and weaknesses of methods for gene network inference. Proc Natl Acad Sci U S A.

[49] De Andrade JP, Park JM, Gu VW, Woodfield GW, Kulak MV, Lorenzen AW (2016). EGFR is regulated by TFAP2C in luminal breast cancer and is a target for Vandetanib. Mol Cancer Ther.

[50] Selamat SA, Chung BS, Girard L, Zhang W, Zhang Y, Campan M (2012). Genome-scale analysis of DNA methylation in lung adenocarcinoma and integration with mRNA expression. Genome Res.

[51] Rubinstein R, Bruckstein AM, Elad M (2010). Dictionaries for sparse representation modeling. Proc IEEE.

[52] Hastie T, Tibshirani R, Friedman J, Franklin J (2005). The elements of statistical learning: data mining, inference and prediction. Math Intell.

[53] Hyvärinen A, Oja E (2000). Independent component analysis: algorithms and applications. Neural Netw.

[54] Liao JC, Boscolo R, Yang Y-L, Tran LM, Sabatti C, Roychowdhury VP (2003). Network component analysis: reconstruction of regulatory signals in biological systems. Proc Natl Acad Sci.

[55] Chang C, Ding Z, Hung YS, Fung PCW (2008). Fast network component analysis (FastNCA) for gene regulatory network reconstruction from microarray data. Bioinformatics..

[56] Boscolo R, Sabatti C, Liao JC, Roychowdhury VP (2005). A generalized framework for network component analysis. IEEE/ACM Transactions Computational Biol Bioinformatics.

[57] Allison DB, Cui X, Page GP, Sabripour M (2006). Microarray data analysis: from disarray to consolidation and consensus. Nat Rev Genet.

[58] Cheng N, Li M, Zhao L, Zhang B, Yang Y, Zheng CH (2020). Comparison and integration of computational methods for deleterious synonymous mutation prediction. Brief Bioinform.

[59] Schaffter T, Marbach D, Floreano D (2011). GeneNetWeaver: in silico benchmark generation and performance profiling of network inference methods. Bioinformatics..

[60] Gama-Castro S, Salgado H, Peralta-Gil M, Santos-Zavaleta A, Muñiz-Rascado L, Solano-Lira H (2011). RegulonDB version 7.0: transcriptional regulation of Escherichia coli K-12 integrated within genetic sensory response units (Gensor units). Nucleic Acids Res.

[61] Harbison CT, Gordon DB, Lee TI, Rinaldi NJ, Macisaac KD, Danford TW (2004). Transcriptional regulatory code of a eukaryotic genome. Nature..

[62] MacIsaac KD, Wang T, Gordon DB, Gifford DK, Stormo GD, Fraenkel E (2006). An improved map of conserved regulatory sites for Saccharomyces cerevisiae. BMC Bioinformatics.

[63] Karolchik D, Baertsch R, Diekhans M, Furey TS, Hinrichs A, Lu Y (2003). The UCSC genome browser database. Nucleic Acids Res.

[64] Jiang C, Xuan Z, Zhao F, Zhang MQ (2007). TRED: a transcriptional regulatory element database, new entries and other development. Nucleic Acids Res.

