# Supplementary material for: Globally learning gene regulatory networks based on hidden atomic regulators from transcriptomic big data
Source: BMC Genomics. 2020 Oct 14;21:711. doi: 10.1186/s12864-020-07079-8 (PMC7559338; doi:10.1186/s12864-020-07079-8)
Supplement: Supplementary file 1 — Additional file 1: SI Notes. SII Notes. SIII Notes. SIV Notes. [file 12864_2020_7079_MOESM1_ESM.zip › supplementary material/SI Notes.docx]

1. **The modified k-SVD dictionary learning algorithm for GRN inference.**

| **Algorithm(sf*k*-SVD)** |
| --- |
| **Input** |
| Gene expression matrix , where *n* is the number of samples and *p* is the number of genes  Scale-free sparsity parameter for gene *i*: *ti*(*i*=1,2,…,*p*)  The number of signals to be mined: *l* |
| **Output** |
| Learned dictionary with each column corresponding to a mined signal or latent regulator (LR).  Coefficient matrix sparse representation of **Y** using |
| **Initialization**  Set , , *S*=1, where **Y**(1:*l*) represents the 1st to *l-th* column of matrix **Y**. |
| **Step-1.** (Sparse coding) Use any pursuit algorithm, *e.g.* Orthogonal Matching Pursuit (OMP), to compute the representations vectors **x***i* for the expression vector of gene *i*, *i.e.*.  *i*=1, 2,…, *p*,  Then replace the *i*-th column of with **x***i* with *i*=1, 2,…,*p*.  **Step-2.** (Codebook Update Stage) For each column *j*=1,2,…,*l* in , update it by   - Define the group of examples that use this atom = with represents the (*j*, )-th entity of matrix - Compute the overall representation error matrix without using this atom, - Restrict by choosing only the columns corresponding to , and obtain that implies a selection of error columns corresponds to genes that use the atom - Apply SVD . Replace the *j-th* column in with the first column of **U**. Replace the coefficient vector to be the first column of **V** multiplied by - Set, as well as *S*=*S*+1   **Step-3.** If *S*>200 or , return to step-1  **Step-4.** Set |

1. **A proof of the convergence of the sfk-svd algorithm.**

**Theorem** In the dictionary learning algorithm, the sequence with *S*=1,2,…is convergent.

**Proof**: In Step-1 of the *flow chart*, the representation error, *i.e.* the Frobenius norm of, is lowered by the update of column vectors in step by step. In Step-2, the representation error is further lowered in SVD step. Thus, we get the conclusion that the number sequence is monotonically decreasing.

In consideration that for all S>0, so the sequence is bounded.

With the analysis above, the sequence is convergent.

1. **Measures used for method evaluation**

Two measures, *i.e.* recovery rate (RR) and positive predictive value (PPV), were firstly adopted to measure the power of recovering ARs from gene expression data. Suppose *k* true regulators with the expression profiles of in the background network and *l* ARs with the virtual expression profiles of , we define

where is an indicator function valued 1 if the condition is true and 0 otherwise.

Another two measures, *i.e.* area under receiver operating characteristic curve(AUROC) and area under precision–recall curve (AUPR), which are derived from the receiver operating characteristic curve [33](#_ENREF_33) and precision–recall (PR) curve plots [34](#_ENREF_34) respectively, were used to assess the performance of correctly recognizing regulatory relationships. In receiver operating characteristic curves, true positive rates (TPRs) are plotted against false positive rates (FPRs) while in precision–recall (PR) curves, precisions, *i.e.*, the fraction of true positives in true and false positives, are plotted against recalls, *i.e.*, the proportion of true positives in true positives and false negatives. Logically, the larger the values of AUROCs/AUPRs, the better the recognition performance for a binary predictor.

1. **Implementation of the four previous methods for reverse engineering GRNs**

Four previous methods, GENIE3 [1](#_ENREF_1), CLR [2](#_ENREF_2) , ARACNe-AP [3](#_ENREF_3) and ARACNE [4](#_ENREF_4), were used for comparison. Both CLR and ARACNE were implemented using the bioconductor R package “minet”, with the DPI parameter of ARACNE varying among 0, 0.05, 0.1 and 0.2. For GENIE3 and ARACNe-AP, we used the R code downloaded from http://www.montefiore.ulg.ac.be/~huynh-thu/GENIE3.html and the jar package downloaded from [https://sourceforge.net/projects](https://sourceforge.net/projects/), respectively.

1. **Simulation data I Generation**

In Simulation data I, we assumed total *k*=50 regulators, *p=*1500 target genes and *n* samples and created the background regulatory network as a connection matrix of which the elements were sampled from standard normal distribution for a regulatory relationship and valued zero otherwise. To mimic the scale-free and sparsity property of real GRNs, we constrained the background network by forcing 2, 3, 4 and 5 regulators from the total *k*=50 regulators to regulate 800, 400, 200 and 100 of the target genes respectively. For the *k* regulators, their expression matrix in the *n* samples was simulated by randomly sampling from standard normal distribution. The observed expression matrix for the target genes was then synthesized according to **Y=DX** plus Gaussian noise. To have different levels of noise, we varied signal-to-noise ratio (*SNR*) among *SNR*=10, 15, 20, 25, 30 or 40. We also varied the number of samples among *n*=20, 30, 50,100 or 200 to investigate the influence of sample size. Overall, totally 30 (6×5) simulation data scenarios of different noise levels and different samples sizes were considered, and in each scenario, 20 random data sets were generated and used for avoiding randomness in evaluation.

1. **The processing procedure of the raw lung cancer data sets**

Three human lung adenocarcinoma (LUAD) data sets were downloaded from GEO database (GSE32863, GSE10072 and GSE7670, <https://www.ncbi.nlm.nih.gov/gds>), which originally consist of the expression levels of ~25,000 probes in 116 samples, those of ~13,000 probes in 107 samples, and those of ~13,000 probes in 54 samples, respectively. We preprocessed the three raw lung cancer datasets as follows: non-specific or noise genes were filtered out using a coefficient of variation (CV) filter with a CV cutoff of 0.05 and the intensities of multiple probes matching a same Entrez ID were averaged as the expression values of the gene. After preprocessing, there were 4771 common genes left for the three data sets. The trimmed three data sets with these common genes were used for analysis.

1. **Permutation test for evaluating the significance of cs of methylation regulation**

We employ a permutation test to estimate the significance of confidence scores for methylation regulation in gene expression. A critical step in the permutation test is to calculate permuted *cs*s that are required to sample from null hypothesis. To make this, all the samples labels of genes expression and methylation levels in the original datasets are randomly shuffled, respectively, and rerun dlGRN.

1. **Figures**

**Figure S1** Changing curves of RMSEs with iterations on the six *n*=20 scenarios of simulation data I with different noise levels, SNR=10 (a), 15(b), 20(c), 25(d), 30(e) and 40(f).

**Figure S2** Changing curves of RMSEs with iterations on the six n=30 scenarios of simulation data I with different noise levels, SNR=10 (a), 15(b), 20(c), 25(d), 30(e) and 40(f).

**Figure S3** Changing curves of RMSEs with iterations on the six n=50 scenarios of simulation data I with different noise levels, SNR=10 (a), 15(b), 20(c), 25(d), 30(e) and 40(f)

**Figure S4** Changing curves of RMSEs with iterations on the six n=100 scenarios of simulation data I with different noise levels, SNR=10 (a), 15(b), 20(c), 25(d), 30(e) and 40(f).

**Figure S5** Changing curves of RMSEs with iterations on the six n=200 scenarios of simulation data I with different noise levels, SNR=10 (a), 15(b), 20(c), 25(d), 30(e) and 40(f).

**Figure S6** Changes of average RRs and average PPVs of the proposed approach with *l* on simulated data I with SNR=10 (a), 15(b), 20(c), 25(d), 30(e) and 40(f).

**Figure S7** 2677-link GRNs inferred by dlGRN and four previous methods on three lung cancer expression data sets, GSE32863, GSE10072 and GSE7670.The network topology is drawn using the Cytoscape software tool.Yellow nodes (rectangles) represent the 55 transcription factors and pink nodes (circles) represent target genes. Node sizes are proportional to the degrees of connections. *γ*: slope of the fitted power-law curve; *AN*: Average number of correctly called TFs *per* target gene; ACC: average clustering coefficient.

**Figure S8** Venn diagrams of inferred edges on the three lung cancer datasets by dlGRN (a) and four previous methods,GENIE3 (b), CLR (c), ARACNe-AP (d) and ARACNE (e).

**Figure S9** Regulators of target gene "ID2" inferred by dlGRN and previous methods, GENIE3, CLR, ARACNe-AP and ARACNE on the lung cancer data sets (*i.e.*, GSE32863, GSE10072 and GSE7670). Blue lines mean true regulations, of which solid and dotted are called and missed respectively, while red dashed lines mean wrongly called regulations.

**Figure S10** Five possible recovered patterns (P1-5) of a CRS, A→B→C.Blue solid arrows represent direct regulations called and red dashed arrows represent missing regulations.

1. **Tables**

**Table S1** The AUROC and AUPR scores of dlGRN (*α*=0.9) and the four previous algorithms on simulation data I.

[See Supplementary material II.]

**Table S2** The AUROC and AUPR scores by dlGRN and four previous GRN inference approaches (*i.e.*, GENIE3, CLR, ARACNe-AP andARACNE) on Simulation data II, E.coli and S.cerevisiae data sets . As the parameter *eps* for ARACNE varied among 0, 0.05, 0.1, and 0.2, only the best results for ARACNE are shown in this table.

| **Method** | | ***Simulation Data II*** | | ***E. coli*** | | ***S.cerevisiae*** | |
| --- | --- | --- | --- | --- | --- | --- | --- |
|  |  | AUROC(%) | AUPR(%) | AUROC(%) | AUPR(%) | AUROC(%) | AUPR(%) |
| GENIE3 | | 81.50 | **28.36** | **71.67** | **2.11** | 52.94 | 0.31 |
| CLR | | 74.34 | 22.63 | 58.72 | 1.12 | 52.43 | 0.22 |
| ARACNe-AP | | 68.19 | 15.59 | 56.55 | 0.61 | 51.64 | 0.02 |
| ARACNE | | 75.72 | 19.12 | 61.66 | 0.80 | 53.01 | 0.22 |
|  | dlGRN (*l*=100) | 82.41 | 22.91 | 67.38 | 1.05 | 53.79 | 0.33 |
|  | dlGRN (*l*=200) | 83.10 | 23.91 | 67.78 | 1.18 | 54.19 | 0.36 |
| *α*=0.3 | dlGRN (*l*=300) | 82.99 | 24.41 | 67.74 | 1.25 | 54.31 | **0.43** |
|  | dlGRN (*l*=400) | 83.12 | 24.35 | 67.50 | 1.30 | 54.18 | 0.36 |
|  | dlGRN (*l*=500) | **83.24** | 24.4 | 67.27 | 1.04 | 54.06 | 0.37 |
|  | dlGRN (*l*=100) | 82.42 | 22.87 | 67.49 | 1.28 | 53.91 | 0.33 |
|  | dlGRN (*l*=200) | 83.08 | 23.93 | 68.22 | 1.35 | 54.37 | 0.36 |
| *α*=0.5 | dlGRN (*l*=300) | 83.04 | 24.43 | 68.41 | 1.44 | **54.49** | 0.41 |
|  | dlGRN (*l*=400) | 83.10 | 24.41 | 68.29 | 1.44 | 54.32 | 0.40 |
|  | dlGRN (*l*=500) | **83.24** | 24.39 | 68.16 | 1.32 | 54.09 | 0.35 |
|  | dlGRN (*l*=100) | 82.40 | 22.92 | 67.36 | 1.28 | 53.74 | 0.37 |
|  | dlGRN (*l*=200) | 83.08 | 23.93 | 68.44 | 1.65 | 54.27 | 0.39 |
| *α*=0.7 | dlGRN (*l*=300) | 83.03 | 24.37 | 68.79 | 1.43 | 54.34 | 0.40 |
|  | dlGRN (*l*=400) | 83.09 | 24.36 | 68.77 | 1.69 | 54.25 | 0.37 |
|  | dlGRN (*l*=500) | 83.23 | 24.42 | 68.84 | 1.41 | 54.08 | 0.40 |
|  | dlGRN (*l*=100) | 82.4 | 22.9 | 67.24 | 0.72 | 53.34 | 0.33 |
|  | dlGRN (*l*=200) | 83.11 | 23.99 | 68.52 | 1.15 | 53.78 | 0.41 |
| *α*=0.9 | dlGRN (*l*=300) | 83.02 | 24.36 | 69.02 | 1.18 | 54.03 | 0.41 |
|  | dlGRN (*l*=400) | 83.10 | 24.36 | 69.10 | 1.41 | 53.85 | 0.38 |
|  | dlGRN (*l*=500) | **83.24** | 24.41 | 69.26 | 1.47 | 53.88 | 0.35 |
|  | dlGRN (*l*=100) | 82.39 | 22.89 | 67.22 | 0.72 | 53.36 | 0.32 |
|  | dlGRN (*l*=200) | 83.10 | 23.99 | 68.50 | 1.15 | 53.77 | 0.40 |
| *α*=1 | dlGRN (*l*=300) | 83.02 | 24.36 | 69.02 | 1.18 | 54.02 | 0.41 |
|  | dlGRN (*l*=400) | 83.11 | 24.41 | 69.11 | 1.36 | 53.83 | 0.38 |
|  | dlGRN (*l*=500) | **83.24** | 24.38 | 69.27 | 1.45 | 53.89 | 0.35 |

**Table S3** Ten genes whose expression levels were predicted by dlGRN to be methylation-regulated in lung cancer and were also previously reported.

| **Gene** | ***cs*** | ***p*-value** | **Reference** |
| --- | --- | --- | --- |
| FCN3 | 0.825 | 0 | Andrade, F.A., Beltrame, M.H., Bini, V.B., Gonçalves, L.B., Boldt, A.B.W. and de Messias-Reason, I.J. (2017) Association of a new FCN3 haplotype with high ficolin-3 levels in leprosy. PLoS Neglected Tropical Diseases, 11, e0005409. |
| SEZ6L2 | 0.735 | 0 | Wang, Q. (2017). CpG methylation patterns are associated with gene expression variation in osteosarcoma. Molecular Medicine Reports, 16, 901-907. |
| SFN | 0.681 | 0 | Shiba-Ishii, A. and Noguchi, M. (2012) Aberrant Stratifin Overexpression Is Regulated by Tumor-Associated CpG Demethylation in Lung Adenocarcinoma. *The American Journal of Pathology*, **180**, 1653-1662. |
| CDO1 | 0.721 | <1e-3 | Deckers, I.A.G., Schouten, L.J., Van Neste, L., van Vlodrop, I.J.H., Soetekouw, P.M.M.B., Baldewijns, M.M.L.L., Jeschke, J., Ahuja, N., Herman, J.G., van den Brandt, P.A. *et al.* (2015) Promoter Methylation of <em>CDO1</em> Identifies Clear-Cell Renal Cell Cancer Patients with Poor Survival Outcome. *Clin. Cancer. Res.* |
| HBB | 0.637 | <1e-3 | Siegfried, Z., Eden, S., Mendelsohn, M., Feng, X., Tsuberi, B.-Z. and Cedar, H. (1999) DNA methylation represses transcription in vivo. 22, 203. |
| CCDC47 | 0.620 | <1e-3 | Sharma, P., Garg, G., Kumar, A., Mohammad, F., Kumar, S.R., Tanwar, V.S., Sati, S., Sharma, A., Karthikeyan, G., Brahmachari, V. *et al.* (2014) Genome wide DNA methylation profiling for epigenetic alteration in coronary artery disease patients. *Gene*, **541**, 31-40. |
| AGR2 | 0.596 | <1e-3 | Sung, H.Y., Choi, E.N., Lyu, D., Park, A.K., Ju, W. and Ahn, J.-H. (2014) Aberrant hypomethylation-mediated AGR2 overexpression induces an aggressive phenotype in ovarian cancer cells. *Oncol. Rep.*, **32**, 815-820. |
| BDNF | 0.591 | <1e-3 | Martinowich, K., Hattori, D., Wu, H., Fouse, S., He, F., Hu, Y., Fan, G. and Sun, Y.E. (2003) DNA Methylation-Related Chromatin Remodeling in Activity-Dependent <em>Bdnf</em> Gene Regulation. *Science*, **302**, 890-893. |
| CDKN2A | 0.552 | <1e-3 | Baylln, S.B., Herman, J.G., Graff, J.R., Vertino, P.M. and Issa, J.-P. (1997) In Vande Woude, G. F. and Klein, G. (eds.), *Adv. Cancer Res.* Academic Press, Vol. 72, pp. 141-196. |
| LGALS4 | 0.615 | <1e-3 | Selamat, S.A., Chung, B.S., Girard, L., Zhang, W., Zhang, Y., Campan, M., Siegmund, K.D., Koss, M.N., Hagen, J.A., Lam, W.L. *et al.* (2012) Genome-scale analysis of DNA methylation in lung adenocarcinoma and integration with mRNA expression. *Genome Res.*, **22**, 1197-1211. |

References

1. Huynh-Thu, V.A., Irrthum, A., Wehenkel, L. & Geurts, P. Inferring Regulatory Networks from Expression Data Using Tree-Based Methods. *Plos One* **5**, e12776 (2010).

2. Faith, J.J. et al. Large-scale mapping and validation of Escherichia coli transcriptional regulation from a compendium of expression profiles. *PLoS Biol.* **5**, 54-66 (2007).

3. Lachmann, A., Giorgi, F.M., Lopez, G. & Califano, A. ARACNe-AP: gene network reverse engineering through adaptive partitioning inference of mutual information. *Bioinformatics* **32**, 2233-2235 (2016).

4. Margolin, A.A. et al. ARACNE: An algorithm for the reconstruction of gene regulatory networks in a mammalian cellular context. *BMC Bioinformatics* **7** (2006).
